# Surfing the clinical trials of mesenchymal stem cell therapy in ischemic cardiomyopathy

**DOI:** 10.1186/s13287-021-02443-1

**Published:** 2021-06-23

**Authors:** Iman Razeghian-Jahromi, Anthony G. Matta, Ronan Canitrot, Mohammad Javad Zibaeenezhad, Mahboobeh Razmkhah, Anahid Safari, Vanessa Nader, Jerome Roncalli

**Affiliations:** 1 Cardiovascular Research Center, Shiraz University of Medical Sciences, Shiraz, Iran; 2 Department of Cardiology, Institute CARDIOMET, University Hospital of Toulouse, Toulouse, France; 3 Faculty of medicine, Holy Spirit University of Kaslik, Kaslik, Lebanon; 4 Shiraz Institute for Cancer Research, School of Medicine, Shiraz University of Medical Sciences, Shiraz, Iran; 5 Stem Cells Technology Research Center, Shiraz University of Medical Sciences, Shiraz, Iran; 6 Faculty of Pharmacy, Lebanese University, Beirut, Lebanon; 7 Service de Cardiologie A, CHU de Toulouse, Hôpital de Rangueil, 1 avenue Jean Poulhès, TSA 50032, 31059 Toulouse Cedex 9, France

**Keywords:** Mesenchymal stem cells, Ischemic cardiomyopathy, Clinical trials

## Abstract

While existing remedies failed to fully address the consequences of heart failure, stem cell therapy has been introduced as a promising approach. The present review is a comprehensive appraisal of the impacts of using mesenchymal stem cells (MSCs) in clinical trials mainly conducted on ischemic cardiomyopathy. The benefits of MSC therapy for dysfunctional myocardium are likely attributed to numerous secreted paracrine factors and immunomodulatory effects. The positive outcomes associated with MSC therapy are scar size reduction, reverse remodeling, and angiogenesis. Also, a decreasing in the level of chronic inflammatory markers of heart failure progression like TNF-α is observed. The intense inflammatory reaction in the injured myocardial micro-environment predicts a poor response of scar tissue to MSC therapy. Subsequently, the interval delay between myocardial injury and MSC therapy is not yet determined. The optimal requested dose of cells ranges between 100 to 150 million cells. Allogenic MSCs have different advantages compared to autogenic cells and intra-myocardial injection is the preferred delivery route. The safety and efficacy of MSCs-based therapy have been confirmed in numerous studies, however several undefined parameters like route of administration, optimal timing, source of stem cells, and necessary dose are limiting the routine use of MSCs therapeutic approach in clinical practice. Lastly, pre-conditioning of MSCs and using of exosomes mediated MSCs or genetically modified MSCs may improve the overall therapeutic effect. Future prospective studies establishing a constant procedure for MSCs transplantation are required in order to apply MSC therapy in our daily clinical practice and subsequently improving the overall prognosis of ischemic heart failure patients.

## Background

Heart injuries resulting in significant morbidity and mortality remain the leading cause of death [1, 2]. Different degrees of myocardial dysfunction and fibrosis were detected in ischemic and non-ischemic cardiomyopathies. Scar tissue formation which also alters the perfusion of adjacent myocardium is the main factor to overcome in ischemic cardiomyopathies [1, 2]. Treatment modalities were largely developed during the last decades focusing on relieving symptoms, preventing disease progression, and improving survival and quality of life [3]. Meanwhile, mesenchymal stem cells (MSCs) therapy has emerged as one of the promising therapeutic approaches allowing myocardial repair and regeneration [4]. Different types of cells such as peripheral blood/circulating progenitor cells, hematopoietic and mesenchymal stromal bone marrow (BM) cells, cardiac stem cells, stem cells, myoblasts, and adpidose tissue-derived cells have been used with hopeful results in variant settings of cardiovascular disorders [5]. The target properties of MSCs are their ability to promote angiogenesis and to differentiate when implanted in the ischemic tissues [6–8]. The safety of MSC therapy is well established and reported in several meta-analysis [9] while its efficacy is still under investigation [3, 10]. Animal models and first clinical trials have shown positive outcomes in terms of left ventricular ejection fraction (LVEF) improvement, scar burden reduction, and better tissue perfusion after myocardial infarction (MI) [7, 11] whereas translation to routine clinical practice is yet to be confirmed. Lastly, the use of MSCs is lacking for available data or expert consensus defining the preferred cell source, delivery route, time for intervention, and cell types. Herein, we review the clinical trials on MSC therapy for ischemic cardiomyopathy knowing that such as promising therapeutic approach may optimize the management, prognosis, quality of life, and survival of numerous patients.

## Origin and type of mesenchymal stem cells

Despite the intensive focus recently made on MSC therapy in variant fields, the best type of stem cells to use is still not defined [12]. MSCs are present, at different levels, in almost all organs of the human body and isolation techniques with in-vitro culture and expansion have been described [12]. Instead, BM is the traditional source of MSCs. BM-MSCs are characterized by their anti-fibrosis, pro-angiogenic and immunomodulatory effects stimulating the reparation and regeneration of damaged myocardium [13]. The limited risk of tumor or ectopic tissue formation and the non-complexity of MSC isolation procedure from the iliac crest are the major advantages [14]. View the large distribution of fat throughout the human body, adipose tissue is considered as another accessible source of MSCs with similar properties to BM-MSCs. Indeed, it is worthy to mention that adipose tissue stromal cells and BM-MSCs share the same safety profile [15]. Lastly, the umbilical cord is respectively a third source of MSCs characterized by a significantly higher capacity of migration and differentiation compared to MSCs derived from the two sources listed above (Table 1) [23].

**Table 1:** Clinical efficacy of MSC therapy: data from clinical trials

Clinical trials	Design	Type of cells, dose, and delivery route	Studied population	Follow-up (months)	Results
Rationale and design of the first randomized, double-blind, placebo-controlled trial of intra-myocardial injection of autologous bone-marrow-derived mesenchymal stromal cells in chronic ischemic heart failure (MSC-HF Trial) [12].	Phase II, single-center, double-blind, randomized, placebo-controlled trial.	- Autologous bone-marrow-derived MSCs. - 12 to 15 injections, of each 0.2 mL stem cell solution or placebo. - Intra-myocardial injection	60 patients with chronic ischemic heart failure randomized in a 2:1.	12	Significant improvements in left ventricular systolic function (↑LVESV, LVEF, SV, and cardiac output)
Intra-myocardial transplantation of mesenchymal stromal cells for chronic myocardial ischemia and impaired left ventricular function: Results of the MESAMI 1 pilot trial [6].	Bicentric pilot study	- Autologous bone marrow-derived mesenchymal stromal cells. - Mean of 61.5 × 10^6^ cells per patient - Intra-myocardial injection	10 patients with chronic myocardial ischemia, LVEF ≤ 35%, and reversible perfusion defects	24	Safety of MSC therapy with potential improvement in cardiac performance, left ventricular remodeling, and clinically functional status.
Intra-myocardial injection of mesenchymal precursor cells and successful temporary weaning from left ventricular assist device support in patients with advanced heart failure: a randomized clinical trial [16].	Randomized phase 2 clinical trial	- Allogenic mesenchymal precursor cells - 150 million cells - Intra-myocardial injection	159 with end-stage heart failure	12	- No improvement in left ventricular recovery - Higher dose producing the greatest improvement in cardiac structure and function
Dose comparison study of allogeneic mesenchymal stem cells in patients with ischemic cardiomyopathy (The TRIDENT Study) [17].	Double-blind randomized clinical trials	- Allogenic bone marrow-derived human MSCs - 20 million versus 100 million cells. - Trans-endocardial injection	30 patients with ischemic cardiomyopathy.	6	Both doses reduced scar size while only high dose increases
A randomized, double-blind, placebo-controlled, dose-escalation study of intravenous adult human mesenchymal stem cells (prochymal) after acute myocardial infarction [18].	Double-blind, randomized, placebo-controlled trial.	- Allogenic mesenchymal stem cells - Dose-ranging (0.5, 1.6, and 5 million cells/kg) - Intravenous administration	53 patients presenting for first myocardial infarction between 1 to 10 days before randomization.	6	Safety of intravenous administration of MSCs after acute myocardial infarction.
Mesenchymal precursor cells as adjunctive therapy in recipients of contemporary left ventricular assist device [19]	Multicenter, double-blind, sham-procedure controlled trial	- Allogenic MPCs. - 25 million of cells injected during left ventricular assist device implantation. - Intra-myocardial injection	30 patients with end-stage heart failure planned to LVAD implantation were randomized 2:1	12	Administration of MPCs appeared to be safe, and there was a potential signal of efficacy
Intravenous allogenic mesenchymal stem cells for nonischemic cardiomyopathy: safety and efficacy results of a phase ii-a randomized trial [20].	Single-blind, placebo-controlled, crossover, randomized phase II-a trial	- Mesenchymal stem cells - 1.5 × 10^6^ cells/kg - Intravenous administration	22 patients with non-ischemic cardiomyopathy with left ventricular ejection fraction.	3	MSC therapy was safe, caused immunomodulatory effects, and was associated with improvements in health status and functional capacity.
Randomized, double-blind, phase I/II study of intravenous allogenic mesenchymal stromal cells in acute myocardial infarction [21].	A phase I/II randomized, double-blind, single-dose study.	- Bone marrow-derived allogenic MSCs (Stempeucel). - 2 million cells/kg - Intravenous	20 patients who had undergone percutaneous coronary intervention for STEMI were randomly assigned (1:1)	24	Stempeucel was safe and well-tolerated when administered intravenously in AMI patients 2 days after percutaneous coronary intervention
Adipose-derived regenerative cells in patients with ischemic cardiomyopathy: the PRECISE Trial [22].	Randomized, placebo-controlled, double-blind trial.	- ADRCs. - 3 escalating doses 0.4×10^6^ ADRCs/kg, 0.8×10^6^ ADRCs/kg, and 1.2×10^6^ ADRCs/kg. -Transendocardial injections.	21 ADRC-treated and 6 control patients with ischemic cardiomyopathy.	36	- Isolation and trans-endocardial injection of autologous ADRCs in no-option patients were safe and feasible. - ADRCs preserve ventricular function, myocardial perfusion, and exercise capacity.
Safety and efficacy of the intravenous infusion of umbilical cord mesenchymal stem cells in patients with heart failure: a phase 1/2 randomized controlled trial (RIMECARD Trial) [23].	Phase 1/2, randomized, double-blind, placebo-controlled clinical trial.	- Allogenic UC-MSCs (Cellistem, Cells for Cells S.A., Santiago, Chile). - 1 × 10^6^ cells/kg - Intravenous infusion	30 patients with heart failure and reduced ejection fraction under optimal medical treatment.	12	- Intravenous infusion of UC-MSCs was safe. - Improvements in left ventricular function, functional status, and quality of life.
Adipose-derived stromal cells for treatment of patients with chronic ischemic heart disease (my stromalcell trial): a randomized placebo-controlled study [24].	Randomized double-blind placebo-controlled.	- ADSCs from the abdomen were culture expanded and stimulated with VEGF-A165. - 10–15 injections of 0.2 mL of ASCs. - A NOGA Myostar® catheter was used for intra-myocardial cells delivery.	60 patients with CCS/NYHA class II-III, left ventricular ejection fraction > 40%, and at least one significant coronary artery stenosis	6	- ADSCs treatment was safe but did not improve exercise capacity compared to placebo.
Cardiopoietic stem cell therapy in heart failure: the C-CURE (cardiopoietic stem cell therapy in heart failure) multicenter randomized trial with lineage-specified biologics [25].	A prospective, multicenter, randomized trial.	- Pre-treated MSCs with cardiogenic cocktail. - An average of 18 injections per patient. - Endo-ventricular injection using the NOGA.	48 patients with stable heart failure (15–40%) and a history of myocardial infarction.	24	- Cardiopoietic stem cell therapy was found feasible and safe with signs of benefit in chronic HF.
Bone marrow-derived mesenchymal stromal cell treatment in patients with severe ischaemic heart failure: a randomized placebo-controlled trial (MSC-HF trial) [26].	Randomized, double-blind, placebo-controlled trial.	- Autologous bone marrow-derived mesenchymal stromal cells. - 10 to 15 injections of 0.2 mL. - Intra-myocardial injection.	60 patients with ischemic heart failure were randomized 2:1	6	-Intra-myocardial injection of autologous MSCs was safe and improved myocardial function in patients with severe ischemic HF.
Cardiopoietic cell therapy for advanced ischaemic heart failure: results at 39 weeks of the prospective, randomized, double-blind, sham-controlled CHART-1 clinical trial [27].	Large randomized, double-blind, sham-controlled multicentric study.	- Autologous cardiopoietic stem cells. - 60 million cells - Intra-myocardial injection	240 patients with chronic HF secondary to ischemic heart disease, reduced LVEF (< 35%), and at high risk for recurrent HF-related events despite optimal medical therapy.	24	Efficacy and safety of autologous cardiopoietic stem cells in the treatment of chronic ischemic HF.
Comparison of allogenic vs autologous bone marrow-derived mesenchymal stem cells delivered by trans-endocardial injection in patients with ischemic cardiomyopathy: the POSEIDON randomized trial [11].	Phase 1/2 randomized comparative trial.	- Autologous versus allogenic MSCs. - 20 million, 100 million, or 200 million cells (5 patients in each cell type per dose level). - Trans-endocardial injection	30 patients with left ventricular dysfunction due to ischemic cardiomyopathy	12	- MSC injection favorably affected patient functional capacity, quality of life, and ventricular remodeling.
Trans-endocardial mesenchymal stem cells and mononuclear bone marrow cells for ischemic cardiomyopathy: the TAC-HFT randomized trial [28].	A phase 1 and 2 randomized, blinded, placebo-controlled trial.	- MSCs and bone marrow mononuclear cells. - 10 injections. - Trans-endocardial administration.	65 patients with ischemic cardiomyopathy and LVEF less than 50% [MSCs (*n* = 19) with placebo (*n* = 11) and BMCs (*n* = 19) with placebo (*n* = 10)].	12	Trans-endocardial stem cell injection with MSCs or BMCs appeared to be safe for patients with chronic ischemic cardiomyopathy and left ventricular dysfunction.

*ADRCs* adipose-derived regenerative cells, *ADSCs* adipose-derived stromal cells, *BMCs* bone-marrow mononucleated cells, *CCS* Canadian Cardiovascular Society, *HF* heart failure, *LVAD* left ventricular assist device, *LVEF* left ventricular ejection fraction, *LVESV* left ventricular end-systolic volume, *MPCs* mesenchymal precursor cells, *MSCs* mesenchymal stem cells, *NYHA* New York Heart Association, *STEMI* ST-elevation myocardial infarction, *SV* systolic volume, *UC-MSCs* umbilical cord-derived mesenchymal stem cells

Overall, BM, adipose tissue, and umbilical cord are the three main niches origins from which MSCs could be isolated. Given that microenvironmental conditions are different among these niches, many elements may subsequently affect MSCs characteristics [14, 29].

## The matter of autogenic vs. allogenic

MSCs are considered immunoprivileged due to the lack of major histocompatibility complex class II and costimulatory factors [1]. Also, it was shown that paracrine signaling of MSCs prevents their destruction by lymphocytes [17]. However, allogenic MSCs may trigger the generation of alloreactive antibodies and possibly are delivered to a lesser extent to the target site than autogenic peers due to the clearance action of the immune system [11].

Older age and coexisting cardiovascular risk factors or comorbidities may negatively affect the function of autologous MSCs [30]. It was conceivable that older subjects have less functional stem cells [31]. Coronary artery disease patients are more likely to have abnormal BM function and accordingly, their BM-MSCs are impaired [32]. A conflicting result was revealed by a study which showed similar expression of cell membrane markers and cell proliferation between young and old donors [33]. Allogenic MSCs would be preferred over autogenic MSCs at least in acute cardiovascular settings for time-consuming concern and immediate availability [18, 34] while autogenic MSCs would be an optimal choice for cases with chronic coronary artery disease [34] even it requires complex manufacturing and shipment logistics. Otherwise, a significant difference in terms of efficacy was noted between autogenic and allogenic MSC therapy. For example, an improvement of LVEF and a reduction in major cardiac events in dilated cardiomyopathy were only observed with allogenic MSC therapy [9, 35]. The great efficacy of allogenic MSC therapy is explained by the lower detected level of stromal cell-derived factor-1α (SDF-1α) compared to autogenic MSCs [36]. SDF-1α inhibits the secretion of nitrotyrosine by endothelial cells and the generation of mitochondrial ROS which play a role in angiogenesis and cellular proliferation [36]. Overall, allogenic MSCs could be prepared from healthy donors as an off-the-shelf agent [18]. Precultured MSCs (allogenic) have some additional advantages such as making ready-to-use differentiated cells [37]. However, it should be noted that fresh (not-cryopreserved) MSCs might be more efficient [34].

## Mechanisms of action of MSC therapy

**Fig. 1:**
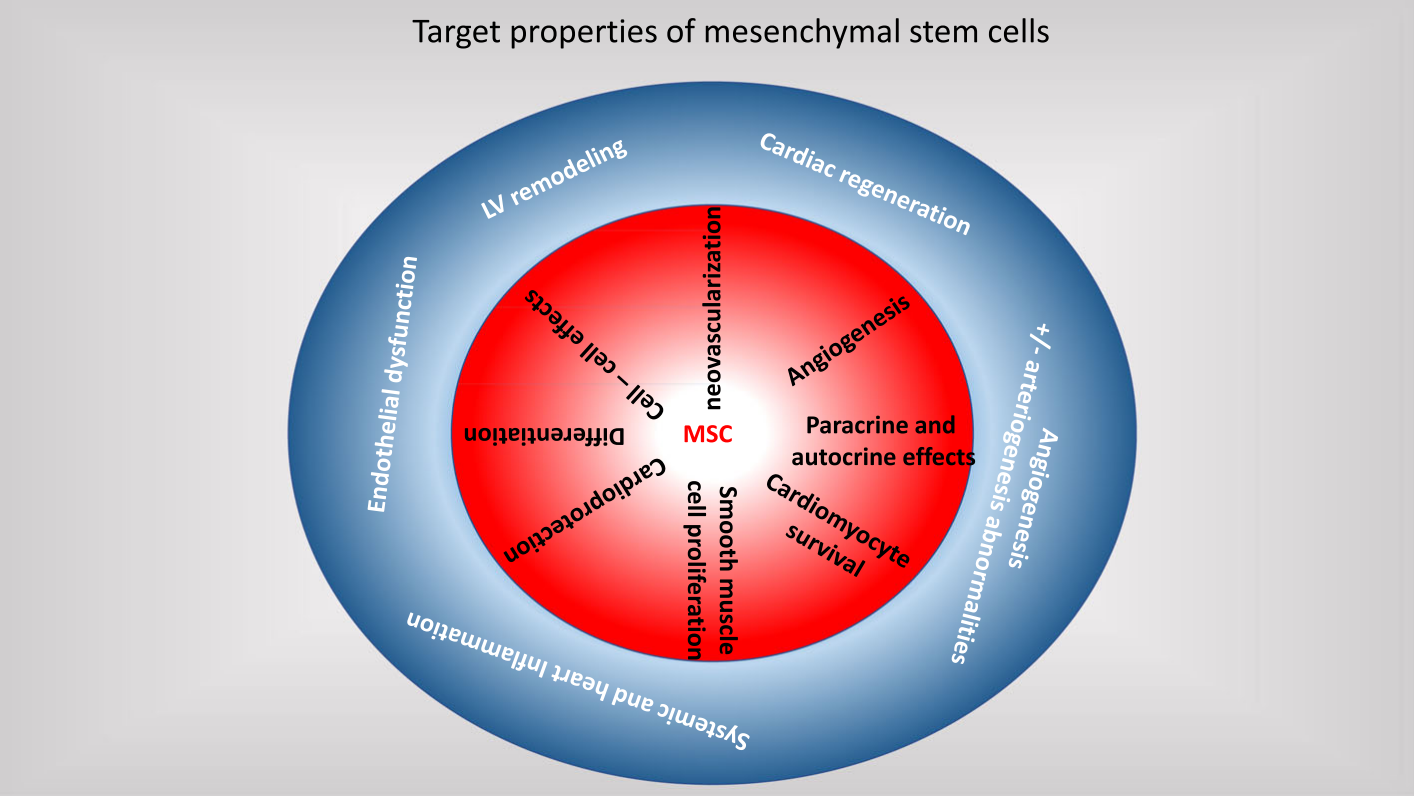
Target properties of mesenchymal stem cells. Relationships between the mechanisms of action of MSC (red circle) and key components of heart failure with reduced ejection fraction (blue circle)

MSCs have anti-fibrotic, anti-inflammatory, anti-apoptotic, immunomodulatory, and pro-angiogenic properties [9]. They play a role in the expression of inflammatory mediators which interfere in homing, chemokine-chemokine receptors interaction, adhesion to endothelial cells, migration into the endothelium, and invasion through the extracellular matrix [38]. MSCs inhibit several immunologic markers liberated during chronic inflammation [39]. In dilated cardiomyopathy patients, MSCs improve cardiac function through restoration of endothelial function which in turn enhances coronary circulation [35]. In ischemic cardiomyopathy patients, anti-fibrotic effect is the most desirable effect as it decreases the scar burden and reverses left ventricular remodeling [11, 40]. MSCs secrete a wide range of molecules with anti-inflammatory and immunomodulatory activities [41]. These molecules have favorable systemic effects, like improving the skeletal muscle performance and organ oxygen delivery especially after an intra-venous administration of MSCs [20]. The main relationships between the mechanisms of MSC and the key components of cardiomyopathies are illustrated in Fig. 1.

The regenerative capacity of MSCs was attributed to several mechanisms, in particular to their paracrine activity. Different growth factors such as SDF-1α, hepatocyte growth factor-1, insulin-like growth factor-1, vascular endothelial growth factor, fibroblast growth factor, and placental growth factor are secreted by MSCs [14]. Also, a wide spectrum of cytokines like angiopoetin-1, matrix metalloproteinase, interleukine-1, interleukine-6, and plasminogen activator is expressed by MSCs [36]. All these agents stimulate cardiomyocyte proliferation in several manners and matrix metalloproteinase secretion leads to fibrosis reduction [14].

The prominent properties of MSCs including participating in the generation of new blood vessels in ischemic tissues and instigating resident cardiac cells resume the interest of MSC therapy in cardiovascular disease (CVD) [6]. One of the unique distinctiveness of MSCs is their ability to transform into endothelial cells, smooth muscle cells and improve the function of resident cardiomyocytes which are the important cell components of the heart [7]. Interestingly, MSCs have a tendency to home, accumulate, and possibly differentiate when locating around the injured microenvironment [8, 10]. MSC therapy in animal models of ischemia resulted in improved ventricular pump function, scar tissue reduction, and neo-angiogenesis after MI [7].

Several preconditioning methods and genetic modifications were suggested to optimize the functional efficacy of transplanted MSCs in vitro and in vivo. Hypoxia preconditioning improves the paracrine effects of MSCs by increasing their metabolic activity, promoting the expression of prion protein, and the secretion of angiogenic and growth factors [42, 43]. In parallel, it decreases the tumorigenic potential of MSCs, the release of lactate dehydrogenase, and the prevalence of aneuploidy in MSCs [44, 45]. All of these result in enhancing the safety and efficacy of stem cell transplantation in different clinical settings. Hu et al. have showed a significantly better improvement in left ventricular function after transplantation of hypoxia pre-treated MSCs compared to normoxia-cultured cells [46]. Also, Han et al. revealed a great recovery of ischemic tissue after injection of hypoxia preconditioning MSCs [47].

Different pharmacological and chemical agents like lenalidomide, vitamin E, sevoflurane, valproic acid, astragaloside IV, apple extract, icariside II, genistein, oxytocin, deferoxamine, atorvastatin, 2,4-dinitrophenol, angiotensin II, angiotensin receptor blockers, low dose lipopolysaccharide, OT, melatonin, rapamycin, all-trans retinoic acid, and polyribocytidylic acid were used to treat MSCs [42]. Focusing on cardiovascular disorders, pre-conditioning of MSCs with deferoxamine and atorvastatin promotes in vivo their homing ability [48] and their long-term survival [49], respectively. Transplantation of pre-treated MSCs with angiotensin II or angiotensin receptor blockers leads in vivo to a better reduction in infarct size and subsequent cardiac fibrosis while it increases the differentiation efficiency of MSCs in vitro [50, 51].

**Fig. 2:**
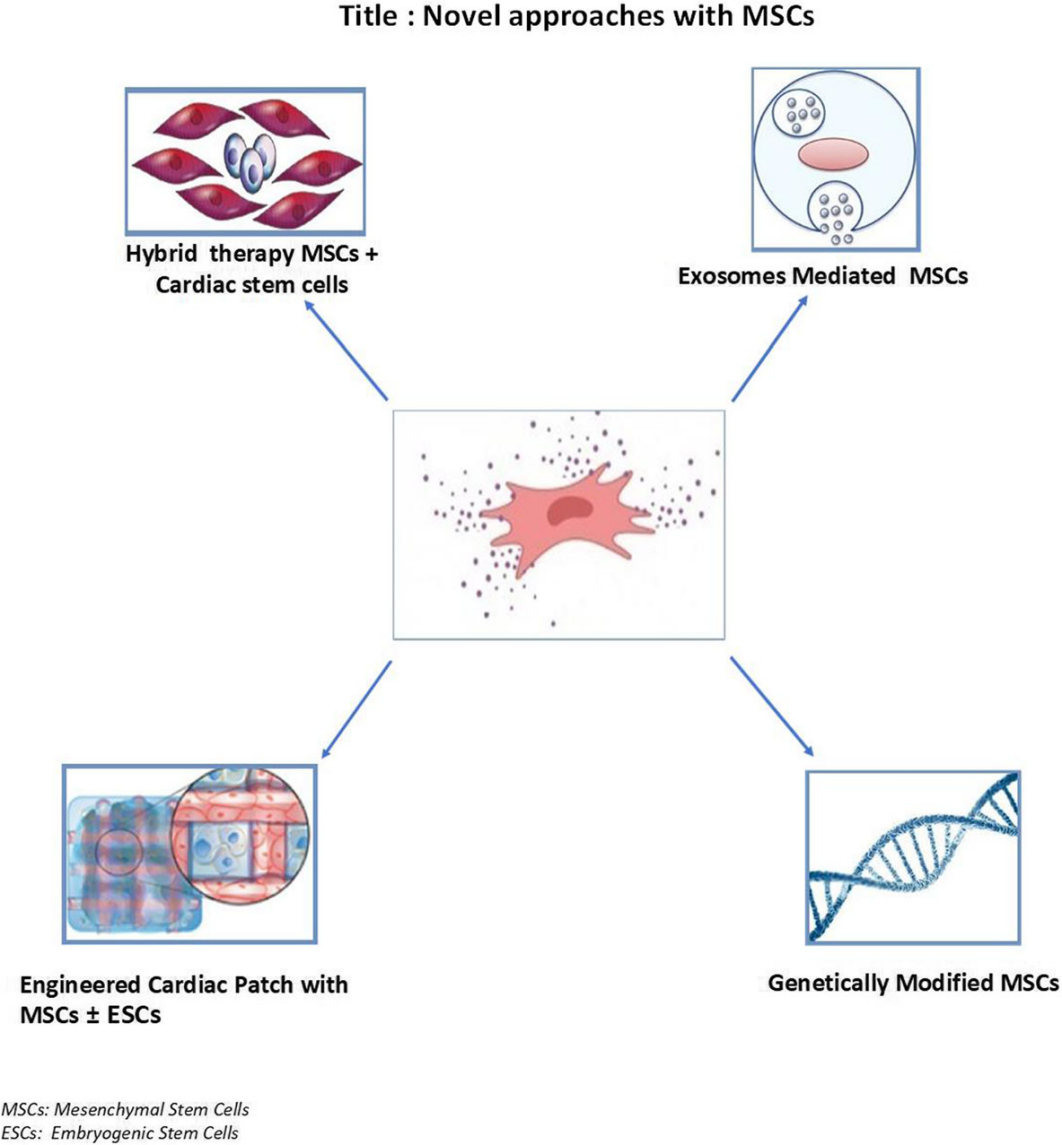
Novel approaches with mesenchymal stem cells. Hybrid therapy combining cells, exosomes mediated MSCs, genetically modified MSCs and Engiennered cardiac patch with MSCs +/− ESCs are future approaches to improve cardiac repair and regeneration. MSCs, mesenchymal stem cells; ESCs, embryogenic stem cells

Lastly, genetic modification of MSCs consists of loading a constructed genetic vector into the MSCs in order to produce or overexpress specific genes aiming to improve their migration, adhesion, survival, and reduce premature senescence. Huang et al. revealed that overexpressing CCR1 increases MSCs viability, migration, engraftment, and capillary density in the infarcted myocardium [52]. Furthermore, the overexpression of integrin and focal adhesion complex by genetically modified MSCs increases by 1.5 times their survival, by fourfold their retention rate, and by 32% their adhesion to ischemic cardiomyocytes when compared to non-modified cells [53]. Also, the transplantation of pre-treated MSCs overexpressing Integrin-linked kinase improves their survival and angiogenic function [54]. Figure 2 showed the novel therapeutic strategies of MSC therapy.

## Clinical efficacy of MSC therapy: data from clinical trials (Table 1)

Positive results have been achieved with MSC therapy in acute and chronic MI in animal models but there are divergent findings from clinical trials [22]. Nonsignificant improvement in LVEF, left ventricular end-systolic volume (LVESV), and left ventricular end-diastolic volume (LVEDV) were reported in the TAC-HFT trial while an increased LVEF with decreased LVESV and no change in LVEDV was seen in the MSC-HF trial. Both trials deployed intra-myocardial injections of autologous BM-MSCs in ischemic HF patients [26, 28].

An infarct size reduction, left ventricular reverse remodeling, improvement of regional myocardial wall contractility, decreasing in end-diastolic and end-systolic volume (LVEDV/LVESV) were shown by Williams et al. [55] in ischemic cardiomyopathy while an ameliorated physical capacity and LVEF were reported by Haack-Sørensen et al. in stable chronic coronary artery disease patients with refractory angina after 12 months of MSC therapy [56]. Also, Lee et al. showed an improvement in myocardial perfusion and global cardiac function after 6 months of injection of BM-MSCs in the culprit coronary artery in ST-elevation myocardial infarction (STEMI) patients with a good safety profile [35]. Instead, Chullikana et al. noted the absence of clinical benefits in STEMI patients after intravenous injection of BM-MSCs [21]. The MSC-HF trial performed on ischemic HF patients showed an improvement of echocardiographic parameters of left ventricular systolic function such as LVESV, LVEF, stroke volume, cardiac output, LV mass, and wall thickness [26]. Thereafter, our MESAMI-1 trial conducted on patients with severe left ventricular dysfunction secondary to coronary artery disease reported an improvement in New York Heart Association (NYHA) functional class, 6-min walk test, and LVEF [6]. The increased LVEF in MESAMI 1 trial was associated to a decrease in summed stress scores (SSS), a cumulative perfusion score, and higher longitudinal strains in correlation with the injected myocardial segments indicating an improved myocardial viability. It is known that the SSS predicts adverse cardiac events in patients with prior MI. In opposition, no improvement was observed by Yau et al., in end-stage HF patients after BM-MSC therapy [16]. Similar findings were observed in the C-CURE trial conducted on patients with stable HF and previous history of MI after endo-ventricular injections of cardiopoietic stem cells derived from cytokine cocktail stimulated-MSCs [25]. Then, a different catheter-based delivery system was used in CHART-1 trial improving the intra-myocardial distribution of stem cells [27]. Ascheim et al have demonstrated the safety and signs of efficacy of intra-myocardial injection of mesenchymal precursor cells in patients with end-stage heart failure and comtemporary left ventricular assist device [19].

Otherwise, trials based on injection of MSCs from adipose tissue-like PRECISE and Athena trials showed respectively an improvement in myocardial mass [22] and treadmill maximum oxygen consumption test [57]. Recently, an improvement in clinical symptoms, physical performance, and quality of life were described in chronic coronary artery disease with refractory angina patients after administration of adipose stem cells stimulated by VEGF-A165 in two current trials conducted in 2017 and 2019 [3, 24]. In a porcine model of chronic myocardial ischemia, intracoronary or intravenous infusion of MSCs from the umbilical cord was associated to endothelial cell differentiation, improved myocardial perfusion, collateral vessel development, LVEF recovery, and reduction in myocardial fibrosis [58]. Thus, the RIMECARD trial based on intravenous administration of MSCs derived from the umbilical cord in patients with HF and reduced ejection fraction described an improvement in LVEF, functional status, and quality of life [23].

Lastly, comparing the efficacy of MSC therapy depending on the source (allogenic versus autogenic): no difference in terms of infarct size reduction and reverse remodeling was observed [28] whereas LVEDV was only improved in the allogenic group and LVEF was markedly increased in this group [9]. Furthermore, the baseline level of tumor necrosis factor α (TNFα) was two times more reduced in the allogenic group [36] in correlation with the lowest level of SDF-1 α. Notably, the improvement in NYHA, 6-min walk test, and Minnesota Living with Heart Failure Questionnaire was firstly noted in the autogenic group [11]. Hypothesis concerning the relationship between the injection frequency, quantity of delivered MSCs, and procedural outcomes were raised by several trials. For example, the TRIDENT trial and a study performed by Perin et al. revealed a parallel correlation between the clinical efficacy and the injected dose [17, 59] while the POSEIDON trial reported a better result associated with the lowest delivered dose [11]. Also, the CHART-1 trial showed a greater reverse remodeling in patients treated with less than 20 injections. This finding could explain that a higher number of injections leads to more myocardial damage and inevitably reduced efficacy [27]. Overall, it seems that the association between dose and efficacy is a matter of optimization, but not an endless endeavor to reach the highest cell quantity. However, some factors like the difference in baseline characteristics of functional cardiac parameters and HF severity among participants may affect the conclusion [17]. Thus, Jian et al have shown that 1 week post MI could be the optimal time for MSCs transplantation exerting the great effect on the improvement of cardiac function, angiogenesis, and apoptosis reduction [60]. Data from clinical trials also suggest a minimal effective dose between 100 and 150 million cells while doses ≤ to 70 million and doses ≥ to 200 million were less effective [61] although in several trials a dose of 60 million of cells was injected.

It is worthy to mention that multiple studies have demonstrated the efficacy of transplantation of embryonic or adult cardiac progenitor cells. An improvement in cardiac function [62, 63], generation of a large number of differentiated cardiomyocytes [64], and reduction in scar size and cardiac remodeling were reported [62, 63]. Indeed, Fernandes et al have revealed a significantly better improvement in cardiac function after transplantation of cardiovascular progenitors from embryonic stem cells than BM mononuclear stem cells [65]. The SCIPIO study was the first clinical trial investigating the therapeutic benefits of autologous cardiac progenitor cells in a clinical setting of ischemic cardiomyopathy, thereby showing an increase in cardiac function parameters with no risk of tumor development at 1 year follow-up [66]. A similar result was found in the CONCERT-HF trial with the combination of mesenchymal and c-kit ^+^ cardiac stem cells [67]. However, we hope to overcome in the near future the issues associated to the application of cardiac progenitor cell therapy, like electrical coupling, long-term integration, and undetermined mechanistic aspects. Moreover, a careful analysis of the trials and the reproducibility of the results in large clinical trials is somewhat expected in the future to demonstrate a true efficacy of such investigations and avoid controversies regarding the cardiac stem cells.

## Limitations of MSC therapy

Numerous hurdles like viability of the transplanted cells and route of administration have hampered the establishment of a generalizable policy for the use of MSC therapy in CVD. The microenvironment of an injured myocardial tissue after acute MI is believed to be detrimental for transplanted cells due to hypoxia and high concentration of free radicals [68]. Intra-coronary infusion dilutes the efficacy of MSC therapy because transplanted cells need to extravasate in order to reach the injured myocardium [69]. Even though cells are bio-chemically able to determine the damaged tissue, there might be a physical barrier (such as an occluded artery) that will prohibit these MSCs to reach the impaired areas [70]. Also, a small minority of intra-coronary injected cells remains in the myocardium while a vast proportion of these cells was found in the systemic blood circulation [71, 72]. These and many other issues need to be scrutinized before using cell-based therapy at the bedside. Clinical benefits of MSC therapy have been evidenced in some clinical trials, but there is a kind of bewilderment, as different studies are not coordinated in terms of efficacy criteria. These subjects may explain why stem cell therapy has not been used in a clinical scale up to now. Otherwise, insufficient long-term survival and integration of transplanted cells with ischemic myocardial tissue is an important concern of regenerative medicine based on stem cell therapy [73]. Another main challenge is the potential cell-to-cell interactions between injected cells and ischemic cardiomyocytes contributing to attenuate engraftment efficiency [74]. Indeed, transplantation of pluripotent stem cells may result in increasing the level of intracellular reactive oxygen species in infarcted cardiomyocytes which are harmful to engraftment survival, thereby inducing cell death by their paracrine effects or through a cell-autonomous manner [75]. Nevertheless, each person reacts in a different way and the outcome of any procedure depends on the body recovery capability.

It is noteworthy to highlight on the availability of multiple protocols of MSCs preparation (isolation, culture, seeding, storage) which could affect the therapeutic properties of these cells, thereby leading to unexpected or reduced outcomes [76]. MSCs could be isolated from the BM by cell-sorting methods or cell-adherence-based methods. The latter is more commonly used, but it collects a non-purified heterogeneous mixture of cells including MSCs, hematopoietic cells, endothelial progenitor cells, and endothelial cells [77]. These contaminating cells affect the required expansion of MSCs and subsequently alter the overall therapeutic result [77]. Prior Ficoll or Percoll density gradient centrifugation isolating mononuclear BM cells from the whole BM cells helps in collecting a larger proportion of homogenous MSCs [78]. Sotiropoulo et al. have demonstrated that Corning flask allows to adhere the largest amount of MSCs compared to other flasks (Falcon, Nunc, Greiner) [79]. A low cell seeding density at 100cells/cm^2^ was associated to faster MSCs proliferation [80]. Also, studies showed that cryopreservation does not impair the main properties of MSCs by reporting a similar biological behavior between fresh and cryopreserved cells [81]. Recently, Panès et al have obtained an approval to commercialize an allogenic expanded adipose stem cells (Cx601) product for the treatment of complex perianal fistulas in Crohn’s disease [82]. Application of this protocol in the cardiac setting could be beneficial and constitutes a step toward defining a standard approach for stem cell preparation.

## Perspective on MSCs

Promising advantages were observed while using stem cell-released exosomes (Fig. 2). Exosomes are small enough to travel throughout the tissue barrier decreasing the potential risks of MSC therapy, like undesired engraftment, ectopic tissue formation, and infusion toxicities due to cell homing and cellular rejection [83, 84]. Recently, Pan et al. have demonstrated protective effects of exosomes mediated miR126 MSCs on endothelial cells against ischemic hypoxia via activating the PI3K/Akt/eNOS pathway and inhibiting cleaved caspase 3, thereby promoting migration, survival, and angiogenic function [85]. The ultimate goal of this area will be the routine use of stem cells for different conditions of heart injury by moving beyond the clinical trials. MSC therapy is an exciting non-pharmacological treatment of HF. The conflicting outcomes and the wide spectrum of used protocols from clinical trials result in a lack of consensus defining the optimal procedural parameters like cell source, type, and delivery system [86]. Otherwise, the different types of HF and subsequent pathophysiological mechanisms are another challenging concern. Despite the evolution of therapeutic strategies to ischemic HF, the general approach remains limited in terms of benefits, survival, and quality of life. MSC therapy is a promising approach for ischemic HF highlighting on the ability of cardiomyocytes to regenerate after myocardial injury. A translation of preliminary clinical trials into clinical practice after providing a baseline uniformed procedure is desirable and may revolutionize the management and overall prognosis of ischemic HF patients.

## Conclusion

MSC therapy has proved its safety and efficacy in different forms of ischemic heart disease. However, existing discrepancies in results among clinical trials have been delaying reaching a consensus and a standardized practical approach. Some issues including, but not limited to, duration of myocardial exposure to ischemic conditions, the effect of host variation on the quality of allogenic versus autologous MSCs, and patient-to-patient variability may affect outcomes of MSC therapy. It remains unclear whether the improvement of myocardial function arises from the cell type (like MSCs in the case of our review) or cell donor origin (allogeneic or autogenic). Conducting comparison head-to-head studies have been initiated on these issues and could determine the main impactful player. Existence of diverse criteria and inconsistent findings make it difficult to conclude yet about MSC therapy in heart diseases.

## Data Availability

Not applicable.

## Abbreviations

BM: Bone marrow
CVD: Cardiovascular disease
HF: Heart failure
LVEF: Left ventricular ejection fraction
LVEDV: Left ventricular end-diastolic volume
LVESV: Left ventricular end-systolic volume
MI: Myocardial infarction
MSCs: Mesenchymal stem cells
NYHA: New York Heart Association
SDF-1α: Stromal cell-derived factor-1α
SSS: Summed stress scores
STEMI: ST-elevation myocardial infarction
TNFα: Tumor necrosis factor α
